# AMPA Receptor Modulation in the Treatment of High-Grade Glioma: Translating Good Science into Better Outcomes

**DOI:** 10.3390/ph18030384

**Published:** 2025-03-08

**Authors:** Daniel P. Radin

**Affiliations:** Stony Brook Medical Scientist Training Program, Renaissance School of Medicine at Stony Brook University, 100 Nicolls Road, Stony Brook, NY 11794, USA; daniel.radin@stonybrookmedicine.edu

**Keywords:** AMPA receptor, BDNF, glutamate, neurogliomal synapse, tumor microenvironment, tumor microtube

## Abstract

Glioblastoma (GB) treatment, despite consisting of surgical resection paired with radiation, temozolomide chemotherapy and tumor-treating fields, yields a median survival of 15–20 months. One of the more recently appreciated hallmarks of GB aggressiveness is the co-opting of neurotransmitter signaling mechanisms that normally sustain excitatory synaptic communication in the CNS. AMPA-glutamate receptor (AMPAR) signaling governs the majority of excitatory synaptic activity in the mammalian brain. AMPAR activation in glioma cells activates cellular pathways that enhance proliferation and invasion and confer resistance to approved GB therapeutics. In addition, this review places a specific emphasis on discussing the redefined GB cytoarchitecture that consists of neuron-to-glioma cell synapses, whose oncogenic activity is driven by AMPAR activation on glioma cells, and the discovery of tumor microtubes, which propagate calcium signals throughout the tumor network in order to enhance resistance to complete surgical resection and radiotherapy. These new discoveries notwithstanding, some evidence suggests that AMPAR activation can produce excitotoxicity in tumor cells. This disparity warrants a closer examination at how AMPAR modulation can be leveraged to produce more durable outcomes in the treatment of GB and tumors in peripheral organs that express AMPAR.

## 1. Introduction

Glutamate receptors in the mammalian brain are divided into ionotropic and metabotropic receptor classes. Ionotropic glutamate receptors are predominantly postsynaptic and are further subdivided into AMPA, NMDA, and Kainate receptors, all of which form transmembrane cation channels that open following glutamate binding [1,2,3,4,5]. Of the three ionotropic glutamate receptors, the AMPA-type glutamate receptor (AMPAR) mediates the majority of fast excitatory synaptic transmission in the mammalian brain [2,6,7,8]. As such, AMPAergic signaling in the CNS modulates a myriad of physiological processes such as respiratory rhythmogenesis [9,10,11,12,13,14,15,16,17,18,19,20], memory consolidation [21,22,23,24,25], neuronal migration, and survival [26,27,28,29,30,31]. Thus, it is not surprising that augmenting AMPAergic signaling has been implicated as a potential treatment for several CNS-based disorders, such as opioid-induced respiratory depression [9,10,14,15,16,17,19], ADHD [19,21,25,32], Alzheimer’s disease [24,33,34,35,36,37,38], Parkinson’s disease [29,39,40,41,42], Huntington’s [43,44], ischemic stroke [45,46] and Rett syndrome [47].

Outside of the CNS, AMPAergic signaling has been shown to be vitally important for the maintenance and normal physiological function of visceral structures such as the pancreas, bone, and skin [48,49]. This is evidenced by the fact that disrupting AMPAergic signaling with specific antagonists perturbs insulin production in islet cells of the pancreas [50]. These findings indicate that glutamatergic signaling governs physiological processes in and outside of the CNS vital to mammalian survival.

It is now becoming more accepted that, in addition to its standard role in multiple physiological systems, AMPAergic signaling is vital for the development, growth, and metastatic dissemination of cancers originating inside and outside of the CNS [51,52,53,54,55,56,57,58,59,60,61,62]. Glioblastoma (GB) is the most common and aggressive primary malignant neoplasm diagnosed in adult patients. GB unfortunately carries a poor prognosis despite multimodal treatment consisting of surgical resection paired with chemotherapy, radiation, and tumor-treating fields [63]. Despite multiple different types of therapy, median survival remains ~20 months [63], suggesting targeted safe therapies are desperately needed for these patients. GB is subdivided into classical, mesenchymal, and proneural subtypes based upon genetic alterations [64]. The purpose of this review is to explore the mechanisms by which AMPAR antagonists abrogate cancer cell proliferation and motility and to discuss translational advancements of drug candidates. The potential interaction between AMPAR antagonists and an evolving treatment landscape for primary or secondary CNS tumors is discussed as is the influence that AMPAR activation has on fostering an increasingly complex GB tumor cytoarchitecture. Furthermore, the paradoxical data demonstrating the oncolytic effects of AMPAR activation [65] and the positive allosteric modulation by ampakines [66] and their disparate mechanisms are also discussed. Finally, outstanding questions that have yet to be fully delineated are put forth, such as how AMPAR modulation may affect anti-tumor immune responses and how surgical resection may alter the glutamatergic milieu of the glioma tumor microenvironment.

## 2. AMPAR Antagonism—Abrogation of Key Survival Pathways

For more than two decades, it has been known that abrogating NMDA or AMPA glutamatergic signaling was oncolytic to an array of cancer cells in vitro, regardless of whether these tumors originated inside or outside of the CNS [51]. Antagonists of either receptor inhibited tumor cell proliferation and motility in a calcium-dependent fashion and sensitized tumor cells to standard of care chemotherapies [51]. These early yet crucial findings paved the way for further exploration of modulating ionotropic glutamate signaling for therapeutic advantage in cancer.

**Figure 1:**
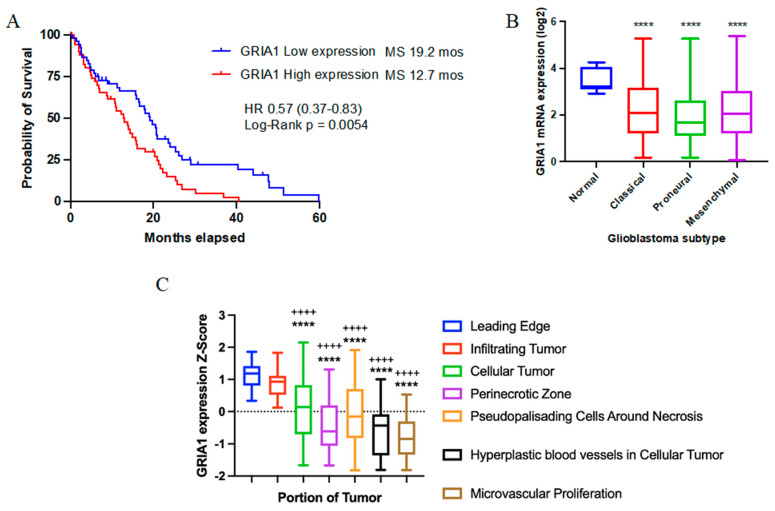
Clinical Correlates of GluR1 (GRIA1) in GB. (**A**) Survival of GB patients stratified by median expression of GRIA1 in IDH wild-type, newly diagnosed, MGMT–methylated GB. (**B**) GRIA1 expression in normal tissue vs. expression in GB subtypes. ANOVA *p* < 0.0001. **** *p* < 0.0001, Dunnett’s multiple comparison test to expression in normal tissue. (**C**) Anatomical distribution of GRIA1 in various GB tumor areas. ANOVA *p* < 0.0001. **** *p* < 0.0001, ++++ *p* < 0.0001, Dunnett’s multiple comparison test to expression in leading edge and infiltrating tumor, respectively. Data for (**A**,**B**) derived from Gliovis and data for (**C**) derived from IvyGAP.

Subsequent studies examined the calcium dependence of the oncolytic effects. For AMPARs, there are four subunits (GluR1–4) that form hetero-tetramers in the cell membrane. For GluR2, a substitution of a critical glutamine (Q) residue to arginine (R) renders the tetramer calcium-impermeable [67,68]. Thus, by using cDNA encoding either GluR2(R) or GluR2(Q), researchers were able to show that the tumorigenic effects of AMPAergic signaling were calcium-dependent in GB [69]. A series of elegant studies described that GB tumor cells predominantly expressed calcium-permeable GluR1 and GluR4 AMPAR subunits and weakly expressed GluR2, if at all. This is in contrast to normal neurons, where GluR2 is expressed in strikingly larger amounts. In primary cultures established from GB biopsies, the AMPARs were found to be functional. By combining AMPA with cyclothiazide (CTZ), an AMPAR positive allosteric modulator, this resulted in a striking increase in cytosolic calcium levels [69], an effect that could be ablated by AMPAR antagonists. Further studies showed that viral infection of the calcium-impermeable subunit GluR2(R) ablated the enhanced motility of GB cells in vitro and abrogated tumor forming ability in vivo. Additional studies illustrated that inducing expression of GluR2(R) ablated the growth of tumors to an extent comparable with administering high doses of an AMPAR antagonist [69] (Table 1). Furthermore, Ishiuchi et al. found that calcium-permeable AMPAR primarily activated akt in glioma cultures [70], which suggests an alternative route for modulating akt in high-grade glioma (Table 1). Akt inhibition by targeting AMPAR may have profound therapeutic implications, as carmustine and temozolomide (TMZ) efficacy increases when akt is inhibited [71,72]. GB patient survival data also suggest that when the AMPAR subunit GluR1 is highly expressed in tumors, patients exhibit a worse prognosis (Figure 1A).

Additional clinical investigations correlate with patient survival data. In a phase 2 clinical trial, adults with newly diagnosed GB were administered the standard of care, surgical resection with radiation and TMZ, along with the experimental AMPAR antagonist Talampanel. In this clinical study, a higher percentage of patients presented with tumors that exhibited unmethylated MGMT promoters compared to historical controls [53], which correlates with reduced response to TMZ [87]. Regardless, median survival time in these patients was 20.3 months versus the historical control median survival of 14.6 months [53]. For patients receiving standard of care plus Talampanel, percentage of patients surviving at 2 years post diagnosis was 41.7% vs. 26%, a statistically significant result. These findings suggest that AMPAR antagonists may enhance the efficacy of GB standard of care without increasing the hematological effects of TMZ [53]. Unfortunately, Talampanel clinical development was discontinued due to its short half-life. However, the preliminary clinical data demonstrates that AMPAR blockage has utility in the clinical setting against GB.

The relationship between AMPAR calcium permeability and oncogenicity was extensively demonstrated in prior literature mainly focusing on GB [69,70]. However, for peripheral tumors expressing functional AMPARs, whether such AMPARs need to be calcium-permeable to drive oncogenic activity is still unclear. For example, studies using non-small cell lung cancer (NSCLC) expressing only the GluR2 subunit indicate that treatment with 2 AMPAR antagonists exerts robust oncolytic activity due to the inhibition of pro-survival kinases such as ERK1/2 and akt [52]. Unfortunately, the authors did not determine whether these GluR2 receptors are calcium-permeable, though they point to the possibility that GluR2 still retains its oncogenic potential regardless of calcium-permeability by interacting with the tyrosine kinase Lyn [88], resulting in Lyn-dependent ERK1/2 activation. An additional study found that siRNA-mediated knockdown of the GluR2 subunit in NSCLC cells resulted in attenuated proliferation and enhanced apoptosis [61].

In other peripheral cancers, AMPARs have been associated with aggressive behavior and resistance to apoptosis, or programmed cell death. Pancreatic cancer, one of the most dismal cancers due to its lack of symptoms and profound, inherent resistance to standard chemotherapies, has been shown to express calcium-permeable AMPARs. Modulation of AMPAR activity, either by genetic means or via the use of two distinct AMPAR antagonists, reduces cell viability and sensitizes cells to the induction of apoptosis [54]. Ectopic overexpression of calcium-permeable GluR3 profoundly accelerated tumor growth in vivo. Similar to how AMPAR activity drives normal pancreatic activity, AMPAR activity seems indispensable for the enhanced proliferative activity, resistance to apoptosis, and augmented motility which serve as hallmark characteristics of pancreatic cancer [54]. AMPAR activation in pancreas tumor cells has also been reported to increase KRAS activity [89], a major driver of pancreatic cancer progression. Several renal carcinoma cell lines overexpress calcium-permeable Glur4 and under express GluR2 [78]. Ablation of calcium uptake with AMPAR antagonist CFM-2 drastically perturbs activation of oncogenic kinases and robustly diminishes renal cancer proliferation and motility (Table 1). These data indicate that peripheral cancers may make use of different AMPAR subunits, though in all published cases, treatment with an AMPAR antagonist results in substantial anti-tumorigenic activity, regardless of the predominant subunit expressed. Experiments with NSCLC indicate that AMPARs possess additional oncogenic activity besides governing calcium uptake in tumor cells. Future research may more directly examine the metabotropic contribution of calcium-permeable and -impermeable AMPARs and the influence of proteins such as Lyn kinase in mediating the oncogenic actions of functional AMPARs.

## 3. Glioma Glutamate Release—Glioma Invasion and Neurodegeneration

One of the hallmarks off GB pathophysiology is the propensity for patients to experience and oftentimes succumb to glioma-associated seizures [90,91,92,93]. Multiple investigations have been undertaken in an effort to understand why these high-grade gliomas are so epileptogenic. Initial reports indicated that glioma cells preferentially secrete the excitatory neurotransmitter glutamate into the extracellular milieu [75,94,95,96,97]. Glutamate secreted by the xCT antiporter [97] has the ability to induce excitotoxic cell death in peritumoral neurons [94] but also fuel the growth and invasion of developing gliomas [75,96]. This disparate effect on cell types in the tumor begs the question as to why glioma cells do not induce their own demise by release of high concentrations of glutamate? While more studies may need to be conducted, available evidence indicates that gliomas express lower levels of AMPAR subunits and thus are less amenable to AMPAR-mediated excitotoxicity than nearby neurons that express higher levels of functional AMPAR [98]. The finding by van Vuurden et al. is also seen in patient data, in which patient tumors, regardless of molecular subtype, express lower levels of AMPAR than non-tumor tissue (Figure 1B). Furthermore, GB tumor anatomical data indicate that the GluR1 subunit is preferentially expressed in the invading portions and edge of gliomas, indicating a predominant role in glioma invasion and tumor cell dissemination (Figure 1C). Finely tuned expression of AMPAR in glioma cells may help these cells in deriving oncogenic activity, such as increased invasive capacity [75] from AMPAR activation, whilst curtailing the antitumor effects of AMPAR excitotoxicity.

Because we have a better understanding of the mechanism by which gliomas induce epileptogenic activity, these processes can be targeted to relieve patients of seizures throughout their disease course and potentially diminish the neurodegenerative effects of rapidly growing gliomas. Perampanel, an FDA-approved AMPAR antagonist for the treatment of seizures, has demonstrated anti-seizure activity in multiple models of glioma. In the C6 glioma model, tumor slices bearing gliomas demonstrated spontaneous recurrent discharges, which were diminished by treatment with Perampanel and the AMPA/kainate receptor antagonist CNQX [83]. A follow-up study by Lange et al. assessed the neuroprotective capacity of Perampanel in the F98 rat glioma model when Perampanel was added to a radiochemotherapy treatment regimen. While Perampanel did not prolong survival as a sole agent, it did reduce epileptogenic activity of glioma-bearing rats to baseline levels. Furthermore, Perampanel rescued glutamate network activity in healthy peritumoral tissue in rats treated with radiochemotherapy [84], suggesting the possibility of neuroprotection by Perampanel against glioma-induced seizures and possibly against the neurotoxic side effects of standard of care therapies used to treat gliomas.

## 4. Neurogliomal Synapses and Tumor Microtubes

Perhaps one of the most exciting developments in the realm of GB research is an increased appreciation for the markedly complex tumor microenvironment fostered by developing high-grade gliomas. The discovery, with prognostic implications, that tumor cells in GB tumors are interconnected was first revealed by Osswald et al. [99], who also found that tumor cells connected with tumor microtubes are better able to resist radiotherapy. Subsequent investigations found that tumor microtubes that extend toward surgically lesioned areas contribute to repopulation of these areas, and that tumor microtube-connected glioma cells exhibit increased resistance to TMZ compared to tumor cells that lack these connections [100]. These seminal publications highlight intercellular communications as profound contributing factors to the poor prognosis of GB. Therapeutic strategies have been investigated to disrupt tumor microtube formation and maintenance, such as microtubule-targeted agents, TGFB inhibitors, and small molecules perturb lysosomal autophagy [101,102,103].

**Figure 2:**
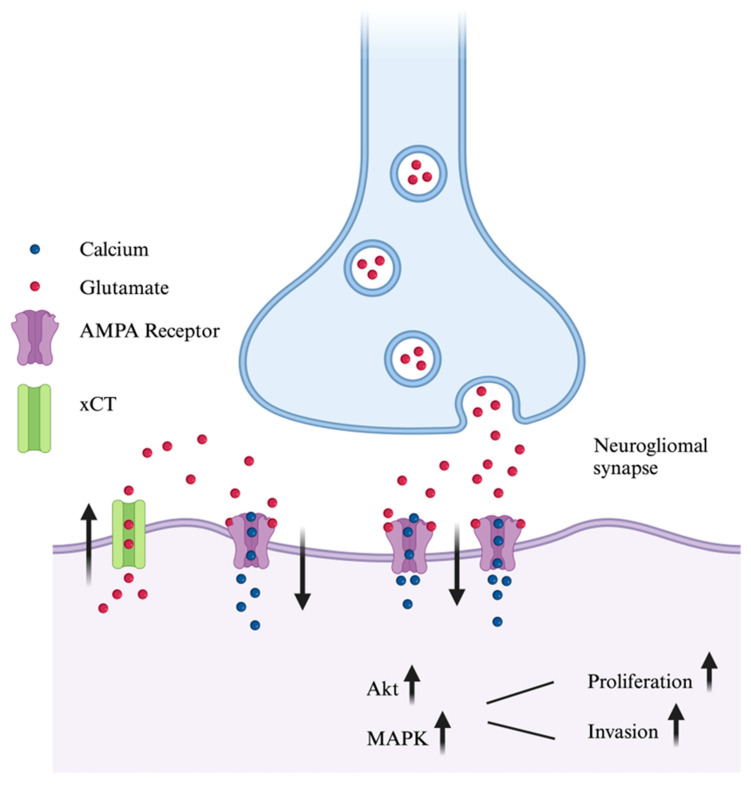
Mechanisms of glioma cell AMPAR activation and downstream effects. Glioma cells can activate AMPAR in an autocrine manner by releasing glutamate which then acts on AMPAR to induce calcium influx. Similarly, peritumoral neurons can synapse on glioma cells, releasing glutamate to activate AMPAR. Calcium influx secondary to AMPAR activation induces activation of Akt and MAPK, which in turn fuels glioma proliferation and invasion.

Adding increased complexity to the GB tumor microenvironment, it was also found that peritumoral neurons synapse onto glioma cells, forming neurogliomal synapses to fuel glioma growth and dissemination [74,82]. These neurogliomal synapses are AMPA glutamatergic in nature and are amenable to AMPAR antagonism in vitro and in vivo [74,82]. Venkataramani et al. found that neurons synapse onto tumor microtubes which generate synchronized calcium transients that are disseminated throughout tumor microtube-connected glioma cells to enhance proliferation and invasion. Treatment of glioma-bearing mice with Perampanel reduced tumor cell proliferation in vivo [82]. Venkatesh et al. also found that Perampanel reduced glioma growth in vivo [74]. Of note, both publications reported that Perampanel was not effective at reducing glioma proliferation in in vitro [74,82], though Venkatesh et al. revealed that when neurons were co-cultured glioma cells, glioma proliferation increased significantly in a manner significantly amenable to AMPAR antagonism [74]. These findings indicate that AMPAR expressed by glioma cells may not be continuously activated in monoculture, and that certain culture or in vivo conditions are necessary to observe oncolytic effects of AMPAR antagonists. Nonetheless, these findings illustrate that AMPAR antagonism may ablate the oncogenic effects of neurogliomal synapse activation (Figure 2), and subsequent propagation of invasive and proliferative signaling cues throughout tumor microtube-connected glioma cells. Whether Perampanel can extend patient survival as an oncolytic agent and whether it can be safely paired with existing standard of care therapies remains to be fully understood.

## 5. AMPAR Activation—Death by Excitotoxicity

Since abrogating AMPAR activity has oncolytic effects in central and peripheral tumors, it stands to reason that AMPAR activation, either by direct agonists or ampakines, should elicit generally oncogenic effects. While this hypothesis is seemingly sensible, this has been shown not to be the case in tumor cells of the CNS and arising from peripheral organs. The first demonstration that AMPAR activation could elicit an anti-cancerous effect was shown using the canonical selective serotonin reuptake inhibitor (SSRI) fluoxetine [65]. 

Fluoxetine was shown previously to selectively execute tumor cells through calcium overload, mitochondrial depolarization, and the induction of intrinsic apoptosis, though the mechanism of this phenomenon was not known. Liu et al. performed a series of studies utilizing several cell surface receptor antagonists in order to hone in on possible secondary targets of fluoxetine. They showed that NBQX, a competitive AMPAR antagonist, was able to perturb fluoxetine-mediated calcium influx and prevent the profound effect of fluoxetine on GB cell viability [65]. The GB cell lines used by the authors intensely expressed GluR1, and that agonism with fluoxetine produced a calcium influx lasting several minutes, inducing subsequent mitochondrial damage, cytochrome C liberation, induction of Caspase-9 activity, and subsequent apoptosis [65]. It is important to consider that in experiments where AMPAR activation augments activity of oncogenic kinases, such increases in activity are usually seen after a several hours of incubation with an agonist. Thus, it seems in this case that the predominant mechanism at play is the one first activated: in this case mitochondrial damage and apoptosis induction occurred before oncogenic kinases could be activated. What is of particular interest is that unlike canonical AMPAR agonists, fluoxetine induced AMPAR calcium currents that did not diminish over time but persisted. This might explain why fluoxetine, and not glutamate, was capable of inducing calcium-dependent excitotoxicity in cancer cells.

Stemming from this work, we recognized that if fluoxetine bound AMPARs at the agonist binding site, its pharmacological actions might be enhanced by concomitant treatment with an ampakine. We first demonstrated that fluoxetine possesses robust oncolytic activity to GB cells, colorectal cancer cells, and pancreas cancer cells in vitro, with all three cell lines having been previously shown to express functional AMPARs. Our subsequent studies demonstrated that CX614, an ampakine that strongly offsets receptor desensitization and prolongs agonist-induced currents [104], also reduces cancer cell viability [66]. Two ampakines that exert little effect on desensitization, CX717 and CX1739, did not alter cancer cell viability [66]. These data demonstrated that offsetting AMPAR desensitization, and in turn inducing excitotoxicity, is necessary to inhibit cancer cell viability whether the cancer cells stem from the CNS or from peripheral organs. Finally, we showed that treating all three cancer cell lines with CX614, then adding fluoxetine to the culture media five minutes later resulted in a synergistic reduction in cell viability [66], providing more evidence that fluoxetine may be inducing its oncolytic effects by AMPAR activation and subsequent excitotoxicity.

## 6. Unanswered Questions

Targeting AMPAR in CNS-associated tumors is made practically and therapeutically possible by the existence of the FDA-approved AMPAR antagonist Perampanel. With drugs like Perampanel, and previously trialed AMPAR antagonists that have been used in proof-of-concept studies, further studies can be undertaken to delineate the possible downstream effects of targeting AMPAR for the treatment of high-grade tumors, regardless of whether they originated inside the CNS. Additional studies can be performed to explore the interaction between AMPAR modulators and approved therapies for the treatment of high-grade tumors such as GB. For example, it is known that Perampanel may augment the effects of TMZ in glioma cells [80,81,105], though no one has published studies on potential interactions between AMPAR antagonism and tumor-treating fields. It is possible, especially in an in vivo context where AMPAR antagonism seems to be more oncolytic, that AMPAR antagonism could synergize with tumor-treating fields. Available evidence suggests that long-term treatment with tumor-treating fields may be enhanced by modalities that diminish Akt activation [106], a protein which is a downstream target of AMPAR in glioma [70]. This may safely extend the therapeutic effects of tumor-treating fields in patients, which principally extended median survival of patients by ~5 months [63]. As GB patients frequently experience seizures, it would be clinically reasonable to start patients on Perampanel when they begin standard post-surgical treatments (TMZ/radiation/tumor-treating fields).

Most of the histological work describing the presence of AMPAR in high-grade tumors has been principally restricted to examination of the presence of certain AMPAR subunits [51,52,69,70]. To date, no work has been done describing the presence of transmembrane AMPAR regulatory proteins (TARPs) in tumor samples. Examining which TARPs, if any, are predominantly expressed alongside AMPAR subunits in tumor cells may have therapeutic implications, as TARP-selective AMPAR antagonists are currently in clinical development [107,108,109,110,111,112,113,114]. Employing AMPAR antagonists that are selective for TARPs expressed by tumor cells and in restricted areas in the CNS may extend therapeutic effects whilst mitigating adverse effects associated with global AMPAR antagonism.

There is a question of how modulating AMPAR will affect local immune function in the tumor microenvironment. As modulating the glioma tumor microenvironment has been explored with PD-1 antagonists [115,116,117,118,119,120] and tumor-treating fields [121], it is worth considering the effects that AMPAR modulation, specifically AMPAR antagonism, might have on the activity of immune cells in the glioma milieu. Preliminary evidence suggests that T-cells express functional AMPAR [122], and that AMPAR antagonism may hinder T-cell activation and proliferation [122,123], thus hindering the overall effectiveness of immune-stimulating therapies.

Finally, regardless of the decades of research that have gone into understanding the pathogenesis of high-grade brain tumors, few groups have successfully been able to model the growth of gliomas following surgical resection. This is in all likelihood due to the inability of researchers to safely resect intracranial tumors from rats and especially mice. Therefore, the alterations of cellular activity that surgical resection has on remnant glioma cells or non-malignant cells that interact with the periphery of the tumor are difficult to parcel out. Recently, however, there has been some progress made in determining how peritumoral astrocytes [124] and glioma and myeloid cells [125] contribute to tumor recurrence and disease lethality. While much of the work may be focusing on proteins secreted in response to surgical resection [124,125], it is possible, if not likely, that glutamate may be secreted in response to surgical intervention. It is widely accepted that shortly after spinal cord injury, glutamate is released in the injured area at toxic levels [126,127], which can exacerbate neurological damage and enhance post-injury deficits [128,129,130]. It could be of potential interest to measure glutamate levels in the resection cavity in preclinical models or measure expression of xCT antiporter as an indirect measurement of glutamate release into the tumor milieu. As autocrine glutamate release can drive glioma cell invasion in an AMPAR-dependent manner [75], it is possible that glioma cells or tumor-associated astrocytes or neurons secrete glutamate which could enhance tumor recurrence and disease progression after surgery. Should this be the case, it would be clinically foreseeable to treat patients with Perampanel prior to and in the immediate post-surgical setting in an effort to hinder oncogenic glutamatergic activity in the resection cavity. As Perampanel has been shown to be well tolerated in the perioperative setting [86], it should be feasible to initiate treatment several days prior to surgery to reach steady-state concentrations at the time of surgical resection.

## 7. Conclusions

Collectively, there exists sufficient data to demonstrate that cancer cells of multiple origins rely on functional AMPAR activity to drive cellular proliferation, migration, and resistance to multiple chemotherapies. Pharmacologically inhibiting AMPAR activity via the use of Talampanel has already shown promise in the clinical setting of newly diagnosed GB [53], highlighting a promising path of future clinical research with the FDA-approved antagonist Perampanel and possibly TARP-specific AMPAR antagonists. Targeting AMPARs could augment the efficacy of chemotherapy, radiation, and tumor-treating fields in high-grade tumors and provide a means of offsetting the oncogenic nature of glioma-specific structures, such as neurogliomal synapses and tumor microtubes.

## Figures and Tables

**Table 1:** Effects of AMPAR modulators on human cancers.

Drug/Molecule	Mechanism	Study	Effects
NBQX	Competitive AMPAR antagonist	[70]	Reduces glioma cell proliferation Reduces Akt activation
		[51]	Reduces medulloblastoma and non-small cell lung cancer proliferation
		[73]	Reduces glioma cell invasion
		[69]	Reduces glioma cell proliferation and increases apoptosis
		[74]	Reduces neuron-stimulated glioma cell proliferation
GYKI 52466	AMPAR negative allosteric modulator	[70]	Reduces glioma cell proliferation Reduces glioma cell migration Reduces Akt activation
		[51]	Reduces medulloblastoma, breast, colon, neuroblastoma, and non-small cell lung cancer proliferation Reduces tumor cell proliferation in a calcium-dependent manner Reduces tumor cell motility
		[75]	Reduces glioma cell invasion
		[76]	Reduces growth of gliomas in brain slices Alleviates peritumoral neuronal cell death
		[52]	Reduces non-small cell lung cancer proliferation Inhibits IGF- and EGF-stimulated lung tumor cell proliferation Decreases ERK1/2 activation Suppresses Cyclin D1 and increased P53 and P21 levels
		[54]	Reduces pancreas tumor cell viability
		[58]	Reduces hypoxia-induced ERK activation in liver cancer cells Reduces hypoxia-induced proliferation of liver cancer cells Induces cell death of renal cancer cells Inhibits invasion and migration of renal cancer cells
SYM2206	AMPAR negative allosteric modulator	[54]	Reduces pancreas tumor cell viability
YM872	Competitive AMPAR antagonist	[69]	Reduces glioma cell proliferation and increases apoptosis Inhibits glioma tumor growth
		[77]	Inhibits glioma tumor growth Reduces glioma tumor vascularity
CFM-2	AMPAR negative allosteric modulator	[52]	Reduces non-small cell lung cancer proliferation Decreases ERK1/2 activation Suppresses Cyclin D1 and increases P53 and P21 levels
		[62]	Reduces non-small cell lung cancer proliferation Reduces Survivin levels
		[78]	Reduces renal cancer cell viability, invasion and Akt activation
Talampanel	AMPAR negative allosteric modulator	[79]	Reduces small cell lung cancer proliferation in vivo and in vitro Reduces MAPK activation in small cell lung cancer
		[53]	Extended median survival of newly diagnosed GB patients to 20.3 months Did not augment the hematological side effects of TMZ
Perampanel	AMPAR negative allosteric modulator	[80]	Reduces glioma cell proliferation Synergistically reduces glioma cell proliferation when paired with TMZ Reduces glioma cell migration
		[81]	Synergistically reduces glioma cell viability when paired with TMZ
		[74]	Reduces glioma tumor cell proliferation in vivo
		[82]	Reduces glioma tumor cell proliferation in vivo
		[83]	Reduces epileptic phenotype in a rat model of glioma
		[84]	Reduces epileptic phenotype in a rat model of glioma
		[85]	Reduces brain tumor-related epileptic seizure frequency in 90% of patients by at least 50%
		[86]	No post-operative seizures were reported in those receiving Perampanel perioperatively
Fluoxetine	Selective serotonin reuptake inhibitor, possible AMPAR agonist	[65]	Induces AMPAR-dependent excitotoxicity in glioma cells in vitro Reduces glioma growth in vivo
CX614	AMPAR positive allosteric modulator	[66]	Reduces viability of glioma, colon, and pancreas tumor cells Synergistically reduces viability of glioma, colon, and pancreas tumor cells when paired with fluoxetine

## References

[1] McFeeters R.L., Oswald R.E. (2004). Emerging structural explanations of ionotropic glutamate receptor function. FASEB J..

[2] Tomita S. (2010). Regulation of ionotropic glutamate receptors by their auxiliary subunits. Physiology.

[3] Traynelis S.F., Wollmuth L.P., McBain C.J., Menniti F.S., Vance K.M., Ogden K.K., Hansen K.B., Yuan H., Myers S.J., Dingledine R. (2010). Glutamate receptor ion channels: Structure, regulation, and function. Pharmacol. Rev..

[4] Sobolevsky A.I. (2013). Structure and Gating of Tetrameric Glutamate Receptors. J. Physiol..

[5] Reiner A., Levitz J. (2018). Glutamatergic Signaling in the Central Nervous System: Ionotropic and Metabotropic Receptors in Concert. Neuron.

[6] Hansen K.B., Yuan H., Traynelis S.F. (2007). Structural aspects of AMPA receptor activation, desensitization and deactivation. Curr. Opin. Neurobiol..

[7] O’Neill M.J., Dix S. (2007). AMPA receptor potentiators as cognitive enhancers. IDrugs.

[8] Milstein A.D., Nicoll R.A. (2008). Regulation of AMPA receptor gating and pharmacology by TARP auxiliary subunits. Trends Pharmacol. Sci..

[9] Ren J., Poon B.Y., Tang Y., Funk G.D., Greer J.J. (2006). Ampakines alleviate respiratory depression in rats. Am. J. Respir. Crit. Care Med..

[10] Ren J., Ding X., Funk G.D., Greer J.J. (2009). Ampakine CX717 protects against fentanyl-induced respiratory depression and lethal apnea in rats. Anesthesiology.

[11] Oertel B.G., Felden L., Tran P.V., Bradshaw M.H., Angst M.S., Schmidt H., Johnson S., Greer J.J., Geisslinger G., Varney M.A. (2010). Selective antagonism of opioid-induced ventilatory depression by an ampakine molecule in humans without loss of opioid analgesia. Clin. Pharmacol. Ther..

[12] Ren J., Ding X., Greer J.J. (2012). Respiratory depression in rats induced by alcohol and barbiturate and rescue by ampakine CX717. J. Appl. Physiol. (1985).

[13] Ren J., Lenal F., Yang M., Ding X., Greer J.J. (2013). Coadministration of the AMPAKINE CX717 with propofol reduces respiratory depression and fatal apneas. Anesthesiology.

[14] Haw A.J., Meyer L.C., Greer J.J., Fuller A. (2016). Ampakine CX1942 attenuates opioid-induced respiratory depression and corrects the hypoxaemic effects of etorphine in immobilized goats (Capra hircus). Vet. Anaesth. Analg..

[15] Dai W., Xiao D., Gao X., Zhou X.B., Fang T.Y., Yong Z., Su R.B. (2017). A brain-targeted ampakine compound protects against opioid-induced respiratory depression. Eur. J. Pharmacol..

[16] Dai W., Gao X., Xiao D., Li Y.L., Zhou X.B., Yong Z., Su R.B. (2019). The Impact and Mechanism of a Novel Allosteric AMPA Receptor Modulator LCX001 on Protection Against Respiratory Depression in Rodents. Front. Pharmacol..

[17] Xiao D., Xie F., Xu X., Zhou X. (2020). The impact and mechanism of ampakine CX1739 on protection against respiratory depression in rats. Future Med. Chem..

[18] Radin D.P., Zhong S., Cerne R., Shoaib M., Witkin J.M., Lippa A. (2024). Low-Impact Ampakine CX1739 Exerts Pro-Cognitive Effects and Reverses Opiate-Induced Respiratory Depression in Rodents. Future Pharmacol..

[19] Radin D.P., Zhong S., Cerne R., Shoaib M., Witkin J.M., Lippa A. (2024). Preclinical characterization of a water-soluble low-impact ampakine prodrug, CX1942 and its active moiety, CX1763. Future Med. Chem..

[20] Rana S., Fusco A.F., Witkin J.M., Radin D.P., Cerne R., Lippa A., Fuller D.D. (2024). Pharmacological modulation of respiratory control: Ampakines as a therapeutic strategy. Pharmacol. Ther..

[21] Zheng Y., Balabhadrapatruni S., Masumura C., Darlington C.L., Smith P.F. (2011). Effects of the putative cognitive-enhancing ampakine, CX717, on attention and object recognition memory. Curr. Alzheimer Res..

[22] Baudry M., Kramar E., Xu X., Zadran H., Moreno S., Lynch G., Gall C., Bi X. (2012). Ampakines promote spine actin polymerization, long-term potentiation, and learning in a mouse model of Angelman syndrome. Neurobiol. Dis..

[23] Radin D.P., Zhong S., Purcell R., Lippa A. (2016). Acute ampakine treatment ameliorates age-related deficits in long-term potentiation. Biomed. Pharmacother..

[24] Mozafari N., Shamsizadeh A., Fatemi I., Allahtavakoli M., Moghadam-Ahmadi A., Kaviani E., Kaeidi A. (2018). CX691, as an AMPA receptor positive modulator, improves the learning and memory in a rat model of Alzheimer’s disease. Iran. J. Basic. Med. Sci..

[25] Tanaka M., Kunugi A., Suzuki A., Suzuki N., Suzuki M., Kimura H. (2019). Preclinical characterization of AMPA receptor potentiator TAK-137 as a therapeutic drug for schizophrenia. Pharmacol. Res. Perspect..

[26] Bahr B.A., Bendiske J., Brown Q.B., Munirathinam S., Caba E., Rudin M., Urwyler S., Sauter A., Rogers G. (2002). Survival signaling and selective neuroprotection through glutamatergic transmission. Exp. Neurol..

[27] Munirathinam S., Rogers G., Bahr B.A. (2002). Positive modulation of alpha-amino-3-hydroxy-5-methyl-4-isoxazolepropionic acid-type glutamate receptors elicits neuroprotection after trimethyltin exposure in hippocampus. Toxicol. Appl. Pharmacol..

[28] Su X.W., Li X.Y., Banasr M., Koo J.W., Shahid M., Henry B., Duman R.S. (2009). Chronic treatment with AMPA receptor potentiator Org 26576 increases neuronal cell proliferation and survival in adult rodent hippocampus. Psychopharmacology.

[29] Jourdi H., Hamo L., Oka T., Seegan A., Baudry M. (2009). BDNF mediates the neuroprotective effects of positive AMPA receptor modulators against MPP+-induced toxicity in cultured hippocampal and mesencephalic slices. Neuropharmacology.

[30] Hachem L.D., Mothe A.J., Tator C.H. (2016). Glutamate Increases In Vitro Survival and Proliferation and Attenuates Oxidative Stress-Induced Cell Death in Adult Spinal Cord-Derived Neural Stem/Progenitor Cells via Non-NMDA Ionotropic Glutamate Receptors. Stem Cells Dev..

[31] Radin D.P., Rogers G.A., Hewitt K.E., Purcell R., Lippa A. (2018). Ampakines Attenuate Staurosporine-induced Cell Death in Primary Cortical Neurons: Implications in the ‘Chemo-Brain’ Phenomenon. Anticancer. Res..

[32] Adler L.A., Kroon R.A., Stein M., Shahid M., Tarazi F.I., Szegedi A., Schipper J., Cazorla P. (2012). A translational approach to evaluate the efficacy and safety of the novel AMPA receptor positive allosteric modulator org 26576 in adult attention-deficit/hyperactivity disorder. Biol. Psychiatry.

[33] Ikonomovic M.D., Mizukami K., Davies P., Hamilton R., Sheffield R., Armstrong D.M. (1997). The loss of GluR2(3) immunoreactivity precedes neurofibrillary tangle formation in the entorhinal cortex and hippocampus of Alzheimer brains. J. Neuropathol. Exp. Neurol..

[34] Yamada K.A. (2000). Therapeutic potential of positive AMPA receptor modulators in the treatment of neurological disease. Expert. Opin. Investig. Drugs.

[35] Ikonomovic M.D., Nocera R., Mizukami K., Armstrong D.M. (2000). Age-related loss of the AMPA receptor subunits GluR2/3 in the human nucleus basalis of Meynert. Exp. Neurol..

[36] Woolley M.L., Waters K.A., Gartlon J.E., Lacroix L.P., Jennings C., Shaughnessy F., Ong A., Pemberton D.J., Harries M.H., Southam E. (2009). Evaluation of the pro-cognitive effects of the AMPA receptor positive modulator, 5-(1-piperidinylcarbonyl)-2,1,3-benzoxadiazole (CX691), in the rat. Psychopharmacology.

[37] Dong Z., Han H., Li H., Bai Y., Wang W., Tu M., Peng Y., Zhou L., He W., Wu X. (2015). Long-term potentiation decay and memory loss are mediated by AMPAR endocytosis. J. Clin. Invest..

[38] Suzuki K., Elegheert J., Song I., Sasakura H., Senkov O., Matsuda K., Kakegawa W., Clayton A.J., Chang V.T., Ferrer-Ferrer M. (2020). A synthetic synaptic organizer protein restores glutamatergic neuronal circuits. Science.

[39] O’Neill M.J., Bleakman D., Zimmerman D.M., Nisenbaum E.S. (2004). AMPA receptor potentiators for the treatment of CNS disorders. Curr. Drug Targets CNS Neurol. Disord..

[40] O’Neill M.J., Murray T.K., Whalley K., Ward M.A., Hicks C.A., Woodhouse S., Osborne D.J., Skolnick P. (2004). Neurotrophic actions of the novel AMPA receptor potentiator, LY404187, in rodent models of Parkinson’s disease. Eur. J. Pharmacol..

[41] O’Neill M.J., Murray T.K., Clay M.P., Lindstrom T., Yang C.R., Nisenbaum E.S. (2005). LY503430: Pharmacology, pharmacokinetics, and effects in rodent models of Parkinson’s disease. CNS Drug Rev..

[42] O’Neill M.J., Witkin J.M. (2007). AMPA receptor potentiators: Application for depression and Parkinson’s disease. Curr. Drug Targets.

[43] Simmons D.A., Rex C.S., Palmer L., Pandyarajan V., Fedulov V., Gall C.M., Lynch G. (2009). Up-regulating BDNF with an ampakine rescues synaptic plasticity and memory in Huntington’s disease knockin mice. Proc. Natl. Acad. Sci. USA.

[44] Simmons D.A., Mehta R.A., Lauterborn J.C., Gall C.M., Lynch G. (2011). Brief ampakine treatments slow the progression of Huntington’s disease phenotypes in R6/2 mice. Neurobiol. Dis..

[45] Clarkson A.N., Overman J.J., Zhong S., Mueller R., Lynch G., Carmichael S.T. (2011). AMPA receptor-induced local brain-derived neurotrophic factor signaling mediates motor recovery after stroke. J. Neurosci..

[46] Clarkson A.N., Parker K., Nilsson M., Walker F.R., Gowing E.K. (2015). Combined ampakine and BDNF treatments enhance poststroke functional recovery in aged mice via AKT-CREB signaling. J. Cereb. Blood Flow. Metab..

[47] Ogier M., Wang H., Hong E., Wang Q., Greenberg M.E., Katz D.M. (2007). Brain-derived neurotrophic factor expression and respiratory function improve after ampakine treatment in a mouse model of Rett syndrome. J. Neurosci..

[48] Skerry T.M., Taylor A.F. (2001). Glutamate signalling in bone. Curr. Pharm. Des..

[49] Skerry T.M., Genever P.G. (2001). Glutamate signalling in non-neuronal tissues. Trends Pharmacol. Sci..

[50] Bertrand G., Gross R., Puech R., Loubatieres-Mariani M.M., Bockaert J. (1992). Evidence for a glutamate receptor of the AMPA subtype which mediates insulin release from rat perfused pancreas. Br. J. Pharmacol..

[51] Rzeski W., Ikonomidou C., Turski L. (2002). Glutamate antagonists limit tumor growth. Biochem. Pharmacol..

[52] Stepulak A., Sifringer M., Rzeski W., Brocke K., Gratopp A., Pohl E.E., Turski L., Ikonomidou C. (2007). AMPA antagonists inhibit the extracellular signal regulated kinase pathway and suppress lung cancer growth. Cancer Biol. Ther..

[53] Grossman S.A., Ye X., Chamberlain M., Mikkelsen T., Batchelor T., Desideri S., Piantadosi S., Fisher J., Fine H.A. (2009). Talampanel with standard radiation and temozolomide in patients with newly diagnosed glioblastoma: A multicenter phase II trial. J. Clin. Oncol..

[54] Ripka S., Riedel J., Neesse A., Griesmann H., Buchholz M., Ellenrieder V., Moeller F., Barth P., Gress T.M., Michl P. (2010). Glutamate receptor GRIA3--target of CUX1 and mediator of tumor progression in pancreatic cancer. Neoplasia.

[55] Iwamoto F.M., Kreisl T.N., Kim L., Duic J.P., Butman J.A., Albert P.S., Fine H.A. (2010). Phase 2 trial of talampanel, a glutamate receptor inhibitor, for adults with recurrent malignant gliomas. Cancer.

[56] Luksch H., Uckermann O., Stepulak A., Hendruschk S., Marzahn J., Bastian S., Staufner C., Temme A., Ikonomidou C. (2011). Silencing of selected glutamate receptor subunits modulates cancer growth. Anticancer. Res..

[57] Oh M.C., Kim J.M., Safaee M., Kaur G., Sun M.Z., Kaur R., Celli A., Mauro T.M., Parsa A.T. (2012). Overexpression of calcium-permeable glutamate receptors in glioblastoma derived brain tumor initiating cells. PLoS ONE.

[58] Hu H., Takano N., Xiang L., Gilkes D.M., Luo W., Semenza G.L. (2014). Hypoxia-inducible factors enhance glutamate signaling in cancer cells. Oncotarget.

[59] Wee S., Niklasson M., Marinescu V.D., Segerman A., Schmidt L., Hermansson A., Dirks P., Forsberg-Nilsson K., Westermark B., Uhrbom L. (2014). Selective calcium sensitivity in immature glioma cancer stem cells. PLoS ONE.

[60] Walczak K., Deneka-Hannemann S., Jarosz B., Zgrajka W., Stoma F., Trojanowski T., Turski W.A., Rzeski W. (2014). Kynurenic acid inhibits proliferation and migration of human glioblastoma T98G cells. Pharmacol. Rep..

[61] Zhang H.Y., Yang W., Lu J.B. (2017). Knockdown of GluA2 induces apoptosis in non-small-cell lung cancer A549 cells through the p53 signaling pathway. Oncol. Lett..

[62] Ruiz D.S., Luksch H., Sifringer M., Temme A., Staufner C., Rzeski W., Marzahn J., Grabarska A., Ikonomidou C., Stepulak A. (2018). AMPA Receptor Antagonist CFM-2 Decreases Survivin Expression in Cancer Cells. Anticancer. Agents Med. Chem..

[63] Stupp R., Taillibert S., Kanner A., Read W., Steinberg D., Lhermitte B., Toms S., Idbaih A., Ahluwalia M.S., Fink K. (2017). Effect of Tumor-Treating Fields Plus Maintenance Temozolomide vs Maintenance Temozolomide Alone on Survival in Patients With Glioblastoma: A Randomized Clinical Trial. JAMA.

[64] Wang Q., Hu B., Hu X., Kim H., Squatrito M., Scarpace L., deCarvalho A.C., Lyu S., Li P., Li Y. (2017). Tumor Evolution of Glioma-Intrinsic Gene Expression Subtypes Associates with Immunological Changes in the Microenvironment. Cancer Cell.

[65] Liu K.H., Yang S.T., Lin Y.K., Lin J.W., Lee Y.H., Wang J.Y., Hu C.J., Lin E.Y., Chen S.M., Then C.K. (2015). Fluoxetine, an antidepressant, suppresses glioblastoma by evoking AMPAR-mediated calcium-dependent apoptosis. Oncotarget.

[66] Radin D.P., Purcell R., Lippa A.S. (2018). Oncolytic Properties of Ampakines In Vitro. Anticancer. Res..

[67] Sans N., Vissel B., Petralia R.S., Wang Y.X., Chang K., Royle G.A., Wang C.Y., O’Gorman S., Heinemann S.F., Wenthold R.J. (2003). Aberrant formation of glutamate receptor complexes in hippocampal neurons of mice lacking the GluR2 AMPA receptor subunit. J. Neurosci..

[68] Isaac J.T., Ashby M.C., McBain C.J. (2007). The role of the GluR2 subunit in AMPA receptor function and synaptic plasticity. Neuron.

[69] Ishiuchi S., Tsuzuki K., Yoshida Y., Yamada N., Hagimura N., Okado H., Miwa A., Kurihara H., Nakazato Y., Tamura M. (2002). Blockage of Ca(2+)-permeable AMPA receptors suppresses migration and induces apoptosis in human glioblastoma cells. Nat. Med..

[70] Ishiuchi S., Yoshida Y., Sugawara K., Aihara M., Ohtani T., Watanabe T., Saito N., Tsuzuki K., Okado H., Miwa A. (2007). Ca2+-permeable AMPA receptors regulate growth of human glioblastoma via Akt activation. J. Neurosci..

[71] Van Meter T.E., Broaddus W.C., Cash D., Fillmore H. (2006). Cotreatment with a novel phosphoinositide analogue inhibitor and carmustine enhances chemotherapeutic efficacy by attenuating AKT activity in gliomas. Cancer.

[72] Zhao W., Zhou L., Zhao W., Yang H., Lu Z., Zhang L., Zhang Y., Xie Y., Lu H., Han W. (2024). The combination of temozolomide and perifosine synergistically inhibit glioblastoma by impeding DNA repair and inducing apoptosis. Cell Death Discov..

[73] Piao Y., Lu L., de Groot J. (2009). AMPA receptors promote perivascular glioma invasion via beta1 integrin-dependent adhesion to the extracellular matrix. Neuro Oncol..

[74] Venkatesh H.S., Morishita W., Geraghty A.C., Silverbush D., Gillespie S.M., Arzt M., Tam L.T., Espenel C., Ponnuswami A., Ni L. (2019). Electrical and synaptic integration of glioma into neural circuits. Nature.

[75] Lyons S.A., Chung W.J., Weaver A.K., Ogunrinu T., Sontheimer H. (2007). Autocrine glutamate signaling promotes glioma cell invasion. Cancer Res..

[76] Savaskan N.E., Seufert S., Hauke J., Trankle C., Eyupoglu I.Y., Hahnen E. (2011). Dissection of mitogenic and neurodegenerative actions of cystine and glutamate in malignant gliomas. Oncogene.

[77] Watanabe T., Ohtani T., Aihara M., Ishiuchi S. (2013). Enhanced antitumor effect of YM872 and AG1296 combination treatment on human glioblastoma xenograft models. J. Neurosurg..

[78] von Roemeling C.A., Radisky D.C., Marlow L.A., Cooper S.J., Grebe S.K., Anastasiadis P.Z., Tun H.W., Copland J.A. (2014). Neuronal pentraxin 2 supports clear cell renal cell carcinoma by activating the AMPA-selective glutamate receptor-4. Cancer Res..

[79] Masumoto N., Kato S., Aichi M., Hasegawa S., Sahara K., Suyama K., Sano A., Miyazaki T., Okudela K., Kaneko T. (2023). AMPAR receptor inhibitors suppress proliferation of human small cell lung cancer cell lines. Thorac. Cancer.

[80] Yagi C., Tatsuoka J., Sano E., Hanashima Y., Ozawa Y., Yoshimura S., Yamamuro S., Sumi K., Hara H., Katayama Y. (2022). Anti-tumor effects of anti-epileptic drugs in malignant glioma cells. Oncol. Rep..

[81] Salmaggi A., Corno C., Maschio M., Donzelli S., D’Urso A., Perego P., Ciusani E. (2021). Synergistic Effect of Perampanel and Temozolomide in Human Glioma Cell Lines. J. Pers. Med..

[82] Venkataramani V., Tanev D.I., Strahle C., Studier-Fischer A., Fankhauser L., Kessler T., Korber C., Kardorff M., Ratliff M., Xie R. (2019). Glutamatergic synaptic input to glioma cells drives brain tumour progression. Nature.

[83] Mayer J., Kirschstein T., Resch T., Porath K., Krause B.J., Kohling R., Lange F. (2020). Perampanel attenuates epileptiform phenotype in C6 glioma. Neurosci. Lett..

[84] Lange F., Hartung J., Liebelt C., Boisseree J., Resch T., Porath K., Hornschemeyer M.F., Reichart G., Sellmann T., Neubert V. (2020). Perampanel Add-on to Standard Radiochemotherapy in vivo Promotes Neuroprotection in a Rodent F98 Glioma Model. Front. Neurosci..

[85] Coppola A., Zarabla A., Maialetti A., Villani V., Koudriavtseva T., Russo E., Nozzolillo A., Sueri C., Belcastro V., Balestrini S. (2020). Perampanel Confirms to Be Effective and Well-Tolerated as an Add-On Treatment in Patients With Brain Tumor-Related Epilepsy (PERADET Study). Front. Neurol..

[86] Kusakabe K., Inoue A., Watanabe H., Nakamura Y., Nishikawa M., Ohtsuka Y., Ogura M., Shigekawa S., Taniwaki M., Kitazawa R. (2023). Perioperative perampanel administration for early seizure prophylaxis in brain tumor patients. Surg. Neurol. Int..

[87] Hegi M.E., Diserens A.C., Gorlia T., Hamou M.F., de Tribolet N., Weller M., Kros J.M., Hainfellner J.A., Mason W., Mariani L. (2005). MGMT gene silencing and benefit from temozolomide in glioblastoma. N. Engl. J. Med..

[88] Hayashi T., Umemori H., Mishina M., Yamamoto T. (1999). The AMPA receptor interacts with and signals through the protein tyrosine kinase Lyn. Nature.

[89] Herner A., Sauliunaite D., Michalski C.W., Erkan M., De Oliveira T., Abiatari I., Kong B., Esposito I., Friess H., Kleeff J. (2011). Glutamate increases pancreatic cancer cell invasion and migration via AMPA receptor activation and Kras-MAPK signaling. Int. J. Cancer.

[90] Riva M., Salmaggi A., Marchioni E., Silvani A., Tomei G., Lorusso L., Merli R., Imbesi F., Russo A., Lombardia Neurooncology G. (2006). Tumour-associated epilepsy: Clinical impact and the role of referring centres in a cohort of glioblastoma patients. A multicentre study from the Lombardia Neurooncology Group. Neurol. Sci..

[91] Bruna J., Miro J., Velasco R. (2013). Epilepsy in glioblastoma patients: Basic mechanisms and current problems in treatment. Expert. Rev. Clin. Pharmacol..

[92] Hertler C., Seystahl K., Le Rhun E., Wirsching H.G., Roth P., Weller M., Gramatzki D. (2022). Improved seizure control in patients with recurrent glioblastoma treated with bevacizumab. Neuro Oncol..

[93] Stritzelberger J., Gesmann A., Fuhrmann I., Uhl M., Brandner S., Welte T.M., Schembs L., Dorfler A., Coras R., Adler W. (2024). The course of tumor-related epilepsy in glioblastoma patients: A retrospective analysis. Epilepsy Behav..

[94] Ye Z.C., Sontheimer H. (1999). Glioma cells release excitotoxic concentrations of glutamate. Cancer Res..

[95] Ye Z.C., Rothstein J.D., Sontheimer H. (1999). Compromised glutamate transport in human glioma cells: Reduction-mislocalization of sodium-dependent glutamate transporters and enhanced activity of cystine-glutamate exchange. J. Neurosci..

[96] Takano T., Lin J.H., Arcuino G., Gao Q., Yang J., Nedergaard M. (2001). Glutamate release promotes growth of malignant gliomas. Nat. Med..

[97] Robert S.M., Buckingham S.C., Campbell S.L., Robel S., Holt K.T., Ogunrinu-Babarinde T., Warren P.P., White D.M., Reid M.A., Eschbacher J.M. (2015). SLC7A11 expression is associated with seizures and predicts poor survival in patients with malignant glioma. Sci. Transl. Med..

[98] van Vuurden D.G., Yazdani M., Bosma I., Broekhuizen A.J., Postma T.J., Heimans J.J., van der Valk P., Aronica E., Tannous B.A., Wurdinger T. (2009). Attenuated AMPA receptor expression allows glioblastoma cell survival in glutamate-rich environment. PLoS ONE.

[99] Osswald M., Jung E., Sahm F., Solecki G., Venkataramani V., Blaes J., Weil S., Horstmann H., Wiestler B., Syed M. (2015). Brain tumour cells interconnect to a functional and resistant network. Nature.

[100] Weil S., Osswald M., Solecki G., Grosch J., Jung E., Lemke D., Ratliff M., Hanggi D., Wick W., Winkler F. (2017). Tumor microtubes convey resistance to surgical lesions and chemotherapy in gliomas. Neuro Oncol..

[101] Horne E.A., Diaz P., Cimino P.J., Jung E., Xu C., Hamel E., Wagenbach M., Kumasaka D., Wageling N.B., Azorin D.D. (2021). A brain-penetrant microtubule-targeting agent that disrupts hallmarks of glioma tumorigenesis. Neurooncol. Adv..

[102] Joseph J.V., Magaut C.R., Storevik S., Geraldo L.H., Mathivet T., Latif M.A., Rudewicz J., Guyon J., Gambaretti M., Haukas F. (2022). TGF-beta promotes microtube formation in glioblastoma through thrombospondin 1. Neuro Oncol..

[103] Radin D.P., Shifman S., Outhwaite I.R., Sharma A., Bases R., Seeliger M.A., Tsirka S.E. (2024). Lucanthone, a Potential PPT1 Inhibitor, Perturbs Stemness, Reduces Tumor Microtube Formation, and Slows the Growth of Temozolomide-Resistant Gliomas In Vivo. J. Pharmacol. Exp. Ther..

[104] Arai A.C., Kessler M., Rogers G., Lynch G. (2000). Effects of the potent ampakine CX614 on hippocampal and recombinant AMPA receptors: Interactions with cyclothiazide and GYKI 52466. Mol. Pharmacol..

[105] Tatsuoka J., Sano E., Hanashima Y., Yagi C., Yamamuro S., Sumi K., Hara H., Takada K., Kanemaru K., Komine-Aizawa S. (2022). Anti-tumor effects of perampanel in malignant glioma cells. Oncol. Lett..

[106] Klein-Goldberg A., Voloshin T., Zemer-Tov E., Paz R., Koren L., Wainer-Katsir K., Volodin A., Koltun B., Brant B., Giladi M. (2021). Activated Phosphoinositide 3-Kinase/AKT/mTOR Signaling Confers Resistance to Tumor Treating Fields (TTFields). Int. J. Radiat. Oncol. Biol. Phys..

[107] Kato A.S., Burris K.D., Gardinier K.M., Gernert D.L., Porter W.J., Reel J., Ding C., Tu Y., Schober D.A., Lee M.R. (2016). Forebrain-selective AMPA-receptor antagonism guided by TARP gamma-8 as an antiepileptic mechanism. Nat. Med..

[108] Gardinier K.M., Gernert D.L., Porter W.J., Reel J.K., Ornstein P.L., Spinazze P., Stevens F.C., Hahn P., Hollinshead S.P., Mayhugh D. (2016). Discovery of the First alpha-Amino-3-hydroxy-5-methyl-4-isoxazolepropionic Acid (AMPA) Receptor Antagonist Dependent upon Transmembrane AMPA Receptor Regulatory Protein (TARP) gamma-8. J. Med. Chem..

[109] Witkin J.M., Schober D.A., Gleason S.D., Catlow J.T., Porter W.J., Reel J., Jin X., Hobbs J., Gehlert D., Gernert D.L. (2017). Targeted Blockade of TARP-gamma8-Associated AMPA Receptors: Anticonvulsant Activity with the Selective Antagonist LY3130481 (CERC-611). CNS Neurol. Disord. Drug Targets.

[110] Lee M.R., Gardinier K.M., Gernert D.L., Schober D.A., Wright R.A., Wang H., Qian Y., Witkin J.M., Nisenbaum E.S., Kato A.S. (2017). Structural Determinants of the gamma-8 TARP Dependent AMPA Receptor Antagonist. ACS Chem. Neurosci..

[111] Witkin J.M., Li J., Gilmour G., Mitchell S.N., Carter G., Gleason S.D., Seidel W.F., Eastwood B.J., McCarthy A., Porter W.J. (2017). Electroencephalographic, cognitive, and neurochemical effects of LY3130481 (CERC-611), a selective antagonist of TARP-gamma8-associated AMPA receptors. Neuropharmacology.

[112] Kato A.S., Witkin J.M. (2018). Auxiliary subunits of AMPA receptors: The discovery of a forebrain-selective antagonist, LY3130481/CERC-611. Biochem. Pharmacol..

[113] Witkin J.M., Ping X., Cerne R., Mouser C., Jin X., Hobbs J., Tiruveedhula V., Li G., Jahan R., Rashid F. (2019). The value of human epileptic tissue in the characterization and development of novel antiepileptic drugs: The example of CERC-611 and KRM-II-81. Brain Res..

[114] Knopp K.L., Simmons R.M.A., Guo W., Adams B.L., Gardinier K.M., Gernert D.L., Ornstein P.L., Porter W., Reel J., Ding C. (2019). Modulation of TARP gamma8-Containing AMPA Receptors as a Novel Therapeutic Approach for Chronic Pain. J. Pharmacol. Exp. Ther..

[115] Schalper K.A., Rodriguez-Ruiz M.E., Diez-Valle R., Lopez-Janeiro A., Porciuncula A., Idoate M.A., Inoges S., de Andrea C., Lopez-Diaz de Cerio A., Tejada S. (2019). Neoadjuvant nivolumab modifies the tumor immune microenvironment in resectable glioblastoma. Nat. Med..

[116] Duerinck J., Schwarze J.K., Awada G., Tijtgat J., Vaeyens F., Bertels C., Geens W., Klein S., Seynaeve L., Cras L. (2021). Intracerebral administration of CTLA-4 and PD-1 immune checkpoint blocking monoclonal antibodies in patients with recurrent glioblastoma: A phase I clinical trial. J. Immunother. Cancer.

[117] Lim M., Weller M., Idbaih A., Steinbach J., Finocchiaro G., Raval R.R., Ansstas G., Baehring J., Taylor J.W., Honnorat J. (2022). Phase III trial of chemoradiotherapy with temozolomide plus nivolumab or placebo for newly diagnosed glioblastoma with methylated MGMT promoter. Neuro Oncol..

[118] Omuro A., Brandes A.A., Carpentier A.F., Idbaih A., Reardon D.A., Cloughesy T., Sumrall A., Baehring J., van den Bent M., Bahr O. (2023). Radiotherapy combined with nivolumab or temozolomide for newly diagnosed glioblastoma with unmethylated MGMT promoter: An international randomized phase III trial. Neuro Oncol..

[119] Kesari S., Wojcinski A., Pabla S., Seager R.J., Gill J.M., Carrillo J.A., Wagle N., Park D.J., Nguyen M., Truong J. (2024). Pre-radiation Nivolumab plus ipilimumab in patients with newly diagnosed high-grade gliomas. Oncoimmunology.

[120] Duerinck J., Lescrauwaet L., Dirven I., Del’haye J., Stevens L., Geeraerts X., Vaeyens F., Geens W., Brock S., Vanbinst A.M. (2024). Intracranial administration of anti-PD-1 and anti-CTLA-4 immune checkpoint-blocking monoclonal antibodies in patients with recurrent high-grade glioma. Neuro Oncol..

[121] Chen D., Le S.B., Hutchinson T.E., Calinescu A.A., Sebastian M., Jin D., Liu T., Ghiaseddin A., Rahman M., Tran D.D. (2022). Tumor Treating Fields dually activate STING and AIM2 inflammasomes to induce adjuvant immunity in glioblastoma. J. Clin. Investig..

[122] Manivannan S., Griffin D. (2010). AMPA receptors are present on mouse lymphocytes and AMPA receptor antagonist, GYKI-52466, inhibits lymphocyte proliferation (50.31). J. Immunol..

[123] Shanker A., de Aquino M.T.P., Hodo T.W., Uzhachenko R. (2020). Glutamate receptor signaling is critical for T cell function and antitumor activity. J. Immunol..

[124] Okolie O., Bago J.R., Schmid R.S., Irvin D.M., Bash R.E., Miller C.R., Hingtgen S.D. (2016). Reactive astrocytes potentiate tumor aggressiveness in a murine glioma resection and recurrence model. Neuro Oncol..

[125] Knudsen A.M., Halle B., Cedile O., Burton M., Baun C., Thisgaard H., Anand A., Hubert C., Thomassen M., Michaelsen S.R. (2022). Surgical resection of glioblastomas induces pleiotrophin-mediated self-renewal of glioblastoma stem cells in recurrent tumors. Neuro Oncol..

[126] Liu D., Thangnipon W., McAdoo D.J. (1991). Excitatory amino acids rise to toxic levels upon impact injury to the rat spinal cord. Brain Res..

[127] McAdoo D.J., Xu G.Y., Robak G., Hughes M.G. (1999). Changes in amino acid concentrations over time and space around an impact injury and their diffusion through the rat spinal cord. Exp. Neurol..

[128] Wrathall J.R., Choiniere D., Teng Y.D. (1994). Dose-dependent reduction of tissue loss and functional impairment after spinal cord trauma with the AMPA/kainate antagonist NBQX. J. Neurosci..

[129] Wrathall J.R., Teng Y.D., Choiniere D. (1996). Amelioration of functional deficits from spinal cord trauma with systemically administered NBQX, an antagonist of non-N-methyl-D-aspartate receptors. Exp. Neurol..

[130] Mu X., Azbill R.D., Springer J.E. (2002). NBQX treatment improves mitochondrial function and reduces oxidative events after spinal cord injury. J. Neurotrauma.

